# Editorialets

**Published:** 1911-07

**Authors:** 


### Editorialets.

Dr. W. J. Mathews, an old resident physician of Austin, died June 5, ult.

Dr. J. H. Reuss of Dallas has gone to Germany for an extended stay, studying in the great medical centers. *Bon voyage.*

The great world famous laparotomist, Dr. Jos. Price of Philadelphia, died at the early age of 58, June 17, ult.

Dios Chemical Co., St. Louis, will send any reader of the "Red Back" a handsome leather-bound prescription and card case arranged to make a carbon duplicate, free on request, naming this notice.

*It is just too hot to write any "able editorials" this month. I'm going camping, to angle for the nimble black bass, and you'll hear from me next month.

FOR SALE.—On account of failing health, a $5000 yearly genito-urinary practice in one of Oklahoma's best cities. A snap for right party with little cash or good paper. Write "Doctor," care of Peerless Drug Co., Enid, Okla.

---

FOR OUR AUGUST NUMBER we have a splendid article by Dr. Bogart, a paper by Dr. Waugh, one by our own Dr. L. H. Reeves of Decatur, one by Dr. Secor of Kerrville and one by Dr. Servoss, and a lot of other good things. The "Red Back" is booming.

---

Ye editor confesses to a rather pronounced liking for preparations fathered by his old friends, Sharp & Dohme, partly because he likes the goods—couldn't keep house without Lapactic Pills, for instance, and partly because we have been warm personal friends for about twenty-five years. The other day we got a neat booklet and a bottle of Phytin Tablets from them. Phytin is the *vegetable* phosphorus as distinguished from the *mineral* phosphorus with which we are all familiar as such and in the form of phosphates, hypophosphites, etc. It is claimed that Phytin is by far the most assimilable and most efficient form of phosphorus. We hope every member of the "Red Back" family will send to Baltimore for a free sample and literature.

